# Inflammatory cues acting on the adult intestinal stem cells and the early onset of cancer (Review)

**DOI:** 10.3892/ijo.2014.2490

**Published:** 2014-06-10

**Authors:** A. DE LERMA BARBARO, G. PERLETTI, I.M. BONAPACE, E. MONTI

**Affiliations:** 1 Biomedical Research Division, Department of Theoretical and Applied Sciences, University of Insubria, Busto Arsizio, Varese, Italy; 2 Department of Basic Medical Sciences, Faculty of Medicine and Health Sciences, Ghent University, Ghent, Belgium

**Keywords:** chronic inflammation, carcinogenesis, cancer stem cells, Paneth cells, colon cancer

## Abstract

The observation that cancer often arises at sites of chronic inflammation has prompted the idea that carcinogenesis and inflammation are deeply interwoven. In fact, the current literature highlights a role for chronic inflammation in virtually all the steps of carcinogenesis, including tumor initiation, promotion and progression. The aim of the present article is to review the current literature on the involvement of chronic inflammation in the initiation step and in the very early phases of tumorigenesis, in a type of cancer where adult stem cells are assumed to be the cells of origin of neoplasia. Since the gastrointestinal tract is regarded as the best-established model system to address the liaison between chronic inflammation and neoplasia, the focus of this article will be on intestinal cancer. In fact, the anatomy of the intestinal epithelial lining is uniquely suited to study adult stem cells in their niche, and the bowel crypt is an ideal developmental biology system, as proliferation, differentiation and cell migration are all distributed linearly along the long axis of the crypt. Moreover, crypt stem cells are regarded today as the most likely targets of neoplastic transformation in bowel cancer. More specifically, the present review addresses the molecular mechanisms whereby a state of chronic inflammation could trigger the neoplastic process in the intestine, focusing on the generation of inflammatory cues evoking enhanced proliferation in cells not initiated but at risk of neoplastic transformation because of their stemness. Novel experimental approaches, based on triggering an inflammatory stimulus in the neighbourhood of adult intestinal stem cells, are warranted to address some as yet unanswered questions. A possible approach, the targeted transgenesis of Paneth cells, may be aimed at ‘hijacking’ the crypt stem cell niche from a status characterized by the maintenance of homeostasis to local chronic inflammation, with the prospect of initiating neoplastic transformation in that site.

## 1. Introduction

Cancer is classically viewed as a genetic disease where the biology, pathophysiology and clinical features of neoplasia are almost entirely defined by events occurring within the genome of the cancer cell. This paradigm has been challenged in recent years, and today cancer is considered as an *ecological* disease, involving a dynamic interplay between malignant and non-malignant cells. Although changes in the genome and epigenome of the cancer cell continues to be considered as essential for the onset and evolution of the disease, there is a growing interest in the heterogeneous array of cells existing within the tumor mass. Pathologists have long recognized that tumors may be densely infiltrated by cells of both the innate and adaptive arms of the immune system, and nowadays it is clear that virtually every neoplastic lesion contains variable quantities of infiltrating immune cells. Whereas in the past the immune infiltrating cells have been thought to reflect an attempt by the immune system to eradicate the growing tumor, in more recent years it is emerging that the immune infiltrate can actually sustain cancer growth, by directly providing growth and mitogenic factors to the tumor mass and by indirectly contributing to the genetic and epigenetic alterations that occur during tumor progression (1).

It is important to remark that this model represents one among many views concerning the liaison between chronic inflammation and cancer. There is clear clinical and epidemiological evidence of the impact of chronic inflammation on cancer. The observation that cancer often arises at sites of chronic inflammation and the indirect evidence of a protective effect of the chronic use of non-steroidal anti-inflammatory drugs (NSAIDs) against both colorectal and prostate cancers are important examples supporting a model whereby inflammatory cells play a causative role in cellular and molecular oncogenesis (2). Moreover, two major pathways leading to inflammation in the cancer microenvironment have been described by Allavena *et al* (3) and Colotta *et al* (4).

In the *intrinsic* pathway, the genetic events in cancer cells may trigger inflammation-related programs that promote the assembly of an inflammatory milieu; conversely, in the *extrinsic* pathway, inflammation may speed up the early neoplastic transformation of normal tissue cells, thus facilitating the oncogenic process in a given tissue. In other words, the trigger of inflammation leading to neoplasia may originate in the tumor stroma (in the extrinsic pathway) or inside the cancer cell itself (in the intrinsic pathway). Finally, chronic inflammation may affect cancer at the different stages of initiation, promotion and progression (5,6).

Our article addresses the role of chronic inflammation in the initiation step; intestinal cancer was chosen as a model, since some of the most convincing examples of carcinogenesis induced by chronic inflammation are seen in the gastrointestinal tract (7).

The anatomy of the intestinal epithelial lining is uniquely suited to study adult stem cells in their niche. The bowel crypt, in particular the Lieberkühn crypt of the small intestine, is indeed an ideal developmental biology system, as proliferation, differentiation and cell migration are distributed linearly along the long axis of the crypt (8).

Crypts are lined with younger transit-amplifying epithelial cells that proliferate and migrate towards the surface of the mucosa and the villi, progressively differentiating and acquiring diverse secretory, enteroendocrine and absorptive functions. Mucosal enterocytes, Paneth and goblet cells derive from stem cells located at the bottom of the crypts, as a result of a finely regulated process involving proliferation, migration and ultimate differentiation of these cells. Interestingly, crypt stem cells are regarded today as the most likely targets of neoplastic transformation in bowel cancer (9,10).

## 2. Epidemiology, histology, pathogenesis and prognosis of colitis-associated colorectal cancer in humans

The general term inflammatory bowel disease (IBD) includes two distinct severe chronic inflammatory conditions of the intestine, Chron’s disease (CD) and ulcerative colitis (UC).

Similar to individuals affected by familial forms of colorectal cancer (CRC) - i.e., adenomatous polyposis and hereditary non-polyposis-associated CRC- IBD patients are at increased risk for developing colitis-associated colorectal cancer (CAC), when compared to sporadic colorectal cancer (CRC) occurring in the general population.

### Epidemiology

As a matter of fact, the exact extent of the risk of developing CRC associated with IBD has not been quantified yet, due to the variable design of the epidemiological studies conducted so far. Nevertheless, there is substantial agreement that the risk of developing CRC in IBD patients exceeds that of subjects without IBD by a factor of approximately 3–5-fold, and that the duration of IBD, its extent, the severity of inflammation, the patient’s gender, the family history of sporadic CRC and the presence of concomitant sclerosing cholangitis are major risk factors, concurring to varying extent to the onset of CRC in patients suffering from IBD (11).

A widely cited meta-analysis of 116 studies performed on a total of 54,478 patients, reports that the estimated cumulative incidence of CRC in UC is 2% after 10 years, 8% after 20 years, and 18% after 30 years of disease (results are from a sub-analysis of 19 studies) (12).

Although risk assessment studies have mostly focused on ulcerative colitis patients, it is emerging that the magnitude of the risk for developing CRC in Crohn’s disease patients is similar to that for UC (13).

Since more recent analyses have claimed lower incidence data (14–17), it has been postulated that the absolute risk of developing CRC is declining, mainly due to increasing worldwide diffusion of endoscopic surveillance protocols.

### Histology

Carcinogenesis in IBD proceeds through increasing grades of dysplasia, generally defined as unequivocally neoplastic but non-invasive epithelium. Remarkably, the sequence of histo-pathological events described in CAC is in sharp contrast with the development of non-IBD-associated familial or sporadic colorectal carcinomas, arising in most cases from single, monofocal adenomatous lesions (18–20).

A widely accepted model of colitis-associated oncogenesis is based on the progressive acquisition of neoplastic features through transition from low-grade dysplasia (LGD) to high-grade dysplasia (HGD) and, ultimately, to multifocal adenocarcinoma. Multifocal CAC lesions emerge from dysplastic mucosa as the result of a field-effect, with a frequency of multiple sychronous lesions ranging between 10 and 30%, compared to 3–5% in the general non-CAC population (18,19).

Dysplastic lesions may be broadly classified as flat or raised, the latter being endoscopically visible. Raised macroscopic lesions may present as resectable adenomas or polyps, or as endoscopically unresectable *dysplasia-associated lesion*-*or*-*mass* (DALM) (19).

Microscopic diagnosis of IBD-associated dysplasia is often difficult, and is characterized by a high degree of inter-observer variability. The histological features of dysplasia include distorted crypts showing cellular atypia and sometimes containing increased numbers of dystrophic goblet cells. In LGD, moderate enlargement, hyperchromasia and mild stratification of the nuclei of crypt epithelial cells are observed. Moreover, polarization is maintained, and nuclei are seen in the basal one-third of epithelial cells. In HGD, nuclear enlargement, hyperchromasia, increased nuclear/cytoplasmic ratio and stratification are more pronounced, there is increasing loss of cellular polarity, and nuclei and mitotic figures are frequently visible in the upper half of epithelial cells (19,21). At the microscopic level, CAC is characterized by a high frequency of mucinous or signet-ring cell phenotypes (22).

### Pathogenesis

Clinically detectable IBD always precedes, sometimes by decades, tumor initiation in CAC. Whereas CAC risk directly correlates with the severity and extent of active inflammatory disease, both familial and sporadic CRC do not arise in the context of preceding inflammation. In other words, whereas in CAC chronic inflammation likely drives the onset of neoplasia from its early stages, in sporadic or familial CRC chronic inflammation may be evoked *by the cancer cell itself*, in a later phase of the neoplastic progression (5,23,24).

Current models of bowel oncogenesis are based on a well established multistep sequence of alterations in oncogenes or tumor suppressor genes occurring within crypt stem cells. Chromosomal and/or microsatellite instability may further contribute to the full neoplastic transformation and clonal expansion of tumor cells (9,25,26).

**Figure 1:**
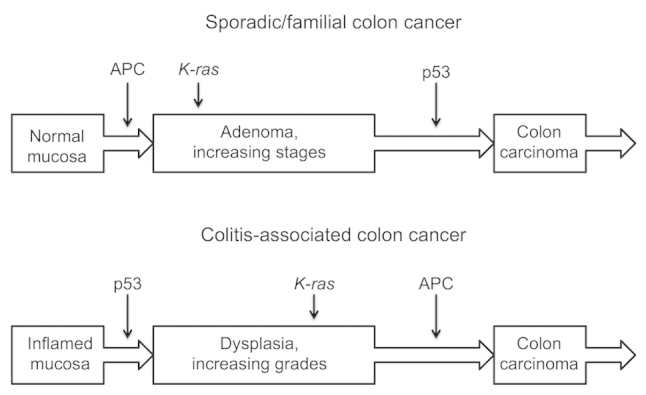
Major steps of sporadic/familial (top) and colitis-associated colon carcinogenesis (bottom). Notably, APC mutations have been shown to appear early in sporadic/familial colonic oncogenesis, and at a later stage in colitis-associated cancer. Conversely, p53 appears to follow an opposite pattern of involvement. Modified from ref. 18.

Interestingly, whereas genes like β-catenin, APC, p53, K-ras and c-src are known to play a pivotal role in tumor cell initiation, promotion and progression, it has become evident that the pattern of sequential activation of these genes in CAC significantly differs from that of familial and sporadic CRC (Fig. 1). The Wnt/β-catenin signaling pathway has been extensively investigated, due to its key role in the renewal of intestinal epithelium and in the fine regulation of normal and malignant cell proliferation. In familial or sporadic CRC, the mutational inactivation of APC, a negative regulator of the Wnt/β-catenin signaling pathway, appears to be an immediate-early event, occurring in over 90% of cancers and triggering the formation of focal early adenomas. Besides APC, GSK3β, a kinase that controls APC, and β-catenin were also found to be mutated, albeit with a lower frequency.

Despite their pivotal role in bowel tumor development, genetic alterations of the Wnt/β-catenin signaling machinery were shown to occur at a late stage of colitis-associated oncogenesis. In particular, APC loss of function, occurring through gene/protein truncation or allelic loss, was shown to take place during the transition from high-grade dysplasia to frank carcinoma (Fig. 1) (18,26).

Remarkably, several lines of evidence suggest that different inflammatory pathways can enhance β-catenin signaling during the early phases of colitis-associated carcinogenesis in the absence of activating mutations.

At variance with the Wnt/β-catenin signaling pathway, which is altered in the early steps of carcinogenesis, *p53* alterations are considered as later events in familiar or sporadic colon oncogenesis, occurring during the final adenoma-carcinoma transition (Fig. 1). Conversely, p53 alterations are apparently taking place in the early stages of the genesis of CAC. It should be stressed that these models are in no way univocal, since some authors describe p53 mutational inactivation as an early step in both sporadic and colitis-associated oncogenesis (27).

Interestingly, both models of colitis-associated and familial/sporadic carcinogenesis involve mutational activation of K-ras and c-src activation as intermediate steps, causing the transition towards increasingly transformed dysplastic or adenomatous lesions, ultimately resulting in the onset of frank carcinoma (26,28).

### Prognosis and management of colon cancers

The prognosis for sporadic CRC and CAC is similar, with a 5-year survival of approximately 50% (11). The diagnosis and grading of colonic dysplasia in endoscopic surveillance biopsies play a key role in the management of patients with IBD, as patients with dysplasia are 3–30-fold as likely to have cancer anywhere in the colorectum, compared to patients not showing dysplastic lesions (29,30). Moreover, it was demonstrated that a diagnosis of CRC is made in 20–50% of IBD patients previously diagnosed with colorectal dysplasia (31,32), and that dysplasia is found together with carcinoma in over 90% of resected surgical specimens (26).

In the presence of HGD, prophylactic proctocolectomy is usually recommended, whereas no consensus has been reached regarding a surgical indication upon detection of LGD. This issue is further complicated by the very high rate of inter-observer variability (50–60%) in the diagnosis of LGD (26,33,34). In any case, a DALM with HGD or LGD, or pancolonic disease, or active disease for over 10 years are considered as indications for proctocolectomy (35).

## 3. Does chronic inflammation play a role in the early stages of colon carcinogenesis? An overview of established and putative mechanisms

The current literature highlights a potential role for chronic inflammation in virtually all steps of carcinogenesis, including tumor initiation and promotion, as well as progression (4,5,23,24). The classical definition of initiation is a set of events that introduce changes into the genome and/or epigenome of an otherwise normal cell, driving its neoplastic transformation. Promotion instead is the action of a substance or stimulus supporting the clonal growth or survival of a previously initiated cell by increasing proliferation or by inhibiting apoptosis; this process is also facilitated by the onset of angiogenesis within the incipient tumor. Finally, progression is the gain of novel molecular and functional features by the *in situ* cancer, that further direct neoplastic growth and tumor mobilization, eventually leading to invasion and metastasis.

Notably, chronic inflammation may extend its influence far beyond the invasion and metastasis steps. In fact, inflammation also drives systemic metabolic alterations such as cachexia (36). Inflammation is traditionally understood to play a role in the tumor promotion step of carcinogenesis. Hence, the preventive effect of NSAIDs on cancer development is likely due to the dampening of chronic inflammation at the stage of tumor promotion (37). In addition, a pivotal role of chronic inflammation in tumor progression is now quite well established (38), for example during the epithelial mesenchymal transition (EMT), which is a key trigger of subsequent invasion and metastasis (39). Yet, an impact of chronic inflammation during the initiation step of carcinogenesis remains experimentally elusive and hence more controversial.

The research group led by Michael Karin has thoroughly investigated the relationship between chronic inflammation and intestinal cancer (5,23,24,27). Their experimental approach has been to alter, by tissue-specific transgenesis, the expression of pivotal players of the inflammatory machinery (e.g., ablation of the NF-κB pathway by conditional knockout of IKKβ) in intestinal epithelial cells (IECs) or in cells of the myeloid lineage. After exposure to a CAC-inducing regimen (see below), significant variations in the rate of cancer onset have been observed in transgenic mice, when compared to parental animals. If transgenesis were found to alter the median number of tumor foci per mouse rather than the tumor median size, it could be implied that inflammation plays a major role in tumor initiation (inflammatory ‘field-effect’) (40); conversely, if a variation in the tumor median size were observed, it might be supposed that inflammation acts mainly as a tumor promoter.

Whether an inflammatory cue that stimulates and sustains proliferation in a cell that is not initiated, but is at risk of neoplastic transformation because of its stemness, should be considered as an initiator or a promoter is a matter of debate. As a matter of fact, experimental approaches quite often do not produce clear-cut results and, accordingly, the distinction between initiation and promotion is not always straightforward.

The mechanisms whereby a state of chronic inflammation could initiate the neoplastic process in the intestine (24) may be approximately classified into four groups. Experimental evidence is available supporting the first two mechanisms, but the last two are rather hypothetical and at present ill-defined.

- Inflammation may act as an initiator of oncogenesis by directly inducing DNA damage and mutagenesis through production of reactive oxygen species (ROS) and reactive nitrogen intermediates (RNI). This issue is well established, since chronic colitis produces local and systemic genotoxic effects, whereby macrophages and neutrophils recruited by inflammation act as the principal sources of ROS and RNI. Within this framework, mutations in the *p53* gene have been identified not only in areas of dysplasia or carcinoma, but also in inflamed, but otherwise normal intestinal mucosa. Moreover, it has been unambiguously demonstrated that prolonged chronic inflammation itself can induce detectable DNA damage and intestinal tumors in murine models, in the absence of exposure to mutagens (24 and references therein).
- Inflammatory signaling may stimulate hyper-proliferation in non-initiated IECs, thus increasing the risk of neoplastic transformation. This is an issue of major concern for our discussion. Notably, inflammatory mediators, such as cytokines IL-1β and TNFα (41–43), or soluble mediators such as prostaglandin E2 (44), may act in some circumstances as growth or survival stimuli. The principal pathways targeted by these agents are Wnt/β-catenin, Akt, NF-κB and STAT3. Interestingly, in most cases the final aftermath of these signal transduction pathways is β-catenin nuclear accumulation even in the absence of APC mutations. A prominent exception is the STAT3 pathway, triggered by IL-6 as well as by other cytokines, where the main effect on IECs is inhibition of apoptosis, which in turn enhances cell survival rates even without involving the Wnt/β-catenin pathway (45,46).
  It remains to be ascertained whether hyper-proliferation of IECs, occurring mainly through a dysregulated Wnt pathway, needs to be continuously stimulated by the pro-inflammatory microenvironment or is rather self-sustained as a consequence of an epigenetic switch. The normal gut microbiota could play an important role in intestinal epigenetic homeostasis by producing high amounts of butyrate, a short chain fatty acid acting mainly through the inhibition of histone deacetylases. In this scenario, a dysbiotic state could cause inadequate butyrate production by the microbiota, thereby facilitating the establishment of pro-cancerous epigenetic tags in IECs (47).
- Chronic intestinal inflammation may start a breakdown of protective intestinal barriers, which causes increased accessibility of IECs to food-borne mutagens. Accordingly, inactivation of Muc2, a major component of the mucus layer in the colon, causes spontaneous intestinal inflammation (48), that progresses to CAC even without further exposure to external carcinogens or mutagens (49). A large body of evidence supports the view that alterations in the gut mucus layer, in the integrity of IEC tight junctions and/or in the gut microbiota may lead to increased susceptibility to intestinal inflammation and cancer (24,50,51).
- Inflammation may cause inactivation of genes encoding DNA proofreading proteins, or enzymes involved in repair mechanisms, such as mismatch repair (MMR). Likewise, inflammation may increase synthesis and activity of activation induced cytidine deaminase AID, which is thought to cause mutations and genetic instability (24).

Although experimental evidence indicating a role for the mechanisms summarized in points i-iv at the very onset of neoplasia has been reported, obtaining a definitive proof of these concepts will be challenging, for reasons that mostly concern the epigenetics of cancer. Evidence accumulated during the past few years indicates that epigenetic changes are strongly associated with cancer development (52). Epigenetic alterations are now recognized to be a driving force of the processes at all steps of carcinogenesis (53). Whether these mechanisms might be involved in very early alterations in cancer, thus anticipating genetic mutations, or whether, most probably, the two types of hereditary alterations concur to cancer development, is still debated. However, it is well known that demethylation of DNA causes mutations and chromosome instability, and aberrant DNA methylation has been observed in many human diseases (54).

Three main epigenetic processes are now recognized to remodel genetic expression programs during development and differentiation: DNA methylation, histone modifications and the activation of the miRNA pathway. Chronic inflammation has been shown to induce epigenetic alterations, and these alterations are also observed during inflammation-induced tumorigenesis, contributing to the processes whereby a normal cell becomes neoplastic (4).

Hypermethylation of the cytosine (5′meC) in CpG islands (CGI) of the promoter regions or in the gene body of tumor suppressors, leading to gene repression, has been observed in several diseases that can degenerate into cancers, including: i) *Helicobacter pylori*-associated gastritis [hypermethylated genes being CDNK2A (p16), FLNC, HAND1, HRASLS, LOX, THBD]; ii) ulcerative colitis (CDNK2A and MyoD1); iii) virus-induced hepatitis (HBV and HCV; CDNK2A, RUNX3); and iv) reflux esophagitis (Barret’s esophagus) (CDNK2A) (for detailed reviews see 55,56). Interestingly, in *Helicobacter pylori*-associated gastritis, the frequency of cells harboring mutations is lower than the frequency of cells with aberrant DNA methylation, suggesting that in gastric mucosae DNA methylation alterations might be important inducers of cell transformation (57). By using glutathione peroxidase double knockout (Gpx1/2-KO) mice as a model of inflammatory bowel disease that predisposes to cancer, Hahn *et al* have shown that 60% of the Polycomb (PcG) target genes that are found to be methylated in tumors were already methylated in the inflamed normal tissue. Moreover, hypermethylation of CGIs in the ileum of Gpx1/2-KO mice is often associated with loss of trimethylation of histone H3 lysine 27 (H3K27) at the same loci. These data suggest that PcG proteins might direct an aberrant inflammatory DNA methylation and histone signature that is observed later in the transformed tissue (57).

A number of studies suggest that cross-signaling between epithelial and stromal cells leads to autocrine and paracrine networks of diffusible factors, resulting in intense cross-talk that contributes to tumor progression (58). Using an animal model in which TGF-β signaling had been deleted in stromal fibroblasts to induce inflammation and DNA damage in the neighbouring epithelia of the forestomach, Achyut *et al* observed loss of transcription of cell cycle-dependent kinase inhibitors p15 and p16 and p21waf1/cip1 hypermethylation (59). Most interestingly, these events were preventable by treating the mice with the selective COX2 inhibitor celecoxib, suggesting a direct role of inflammation in the silencing process.

Although overexpression of IL-1β has been found to be associated with an increased risk of human gastric cancer (60–62), and although upregulation of cytokines have been found in gerbil gastric mucosae infected with *H. pylori* (Cxcl2, IL-1β, Nos2 and TNF) and in human hepatitis and ulcerative colitis (TGF-β, IL-10, TNFα, IL-1β, Nos2) (56,63,64), the mechanism by which cytokine signaling is able to produce epigenetic changes is not yet fully understood.

Global DNA hypomethylation is also observed during tumor induction following inflammation. Repetitive sequences of the genome, such as LINE1, Alu and Satα, amounting to over 40% of the human genome and often used as surrogate markers of genome-wide DNA methylation, are induced to lose 5′meC in gastric mucosae of individuals affected by *H. pylori* infection (65).

Finally, another point of major concern is the role played by hypoxia and hypoxia-induced transcription factors (HIFs) in both models of colon carcinogenesis, i.e., CRC and CAC. Chronic hypoxia regularly occurs in solid tumors, including CRC and CAC; in addition, increases in HIF-dependent signaling can also ensue from activation of oncogenic pathways, including the Wnt/β-catenin, Ras/Raf/MAPK and PI3K/Akt/mTOR pathways, as well as from tumor suppressor gene silencing (66,67). On the other hand, a chronic increase in HIF signaling in colon epithelial cells has been reported to trigger an inflammatory response through direct activation of genes encoding pro-inflammatory cytokines (68,69) and through extensive cross-talk with inflammatory transcription factors, such as STAT3 and NFκB (70). Conversely, an inflammatory microenvironment can sustain high levels of HIF signaling through both oxygen-dependent and -independent mechanisms (71–74). Hypoxic adaptation has long been acknowledged as a major driving force for tumor progression (75), but its role in tumor initiation is still debated. While HIF activation has been reported to lead to acquisition of stem cell-like properties by tumor cells (76), hard evidence for a similar role in non-initiated cells is elusive. However, hypoxia-induced epigenetic silencing (via colonic expression of the transcriptional repressor DEC-1) leading to MMR deficiency and genomic instability has been demonstrated in a mouse IBD model (77), suggesting that HIF-dependent gene expression might also impact on the initiation phase.

In conclusion, chronic inflammation can produce profound alterations in the epigenome in normal tissues that might be causative of neoplastic transformation.

## 4. Intestinal stem cells and their possible involvement in the early onset of bowel cancer

The different regions of the epithelial monolayer lining the gut mucosa display correspondingly different anatomo-functional features and cell composition. Whereas the epithelium of the small intestine is organized into large numbers of self-renewing crypt-villus units, the surface of the large intestine still retains the crypts, yet it is devoid of villi. In both the small and the large intestine crypts are lined with transit-amplifying TA cells which proliferate and migrate towards the lumen surface of the mucosa, progressively differentiating into four main types of post-mitotic cells. Mucosal enterocytes, enteroendocrine cells, goblet and Paneth cells derive from stem cells located at the bottom of the crypts, as a result of a finely regulated process involving proliferation, migration and ultimate differentiation into post-mitotic cells. Of the utmost relevance to this context, Paneth cells are normally found exclusively in the small intestine, and are absent from all other parts of the gastrointestinal tract (78).

The intestinal epithelium represents the most vigorously renewing tissue in adult mammals. Since the unique anatomy of the intestinal crypt epithelium makes it one of the most accessible models for the study of adult stem cell biology, the research on this subject started almost four decades ago (8). However, the stem cells that fuel the gut self-renewal process have been identified only recently. In 2007 Barker *et al* identified Lgr5 as putative marker of crypt stem cells. Lgr5 is an orphan receptor of unknown function, and is targeted by the Wnt signaling pathway (79).

Through an elegant series of lineage tracing experiments, using a knocked-in, tamoxifen-inducible Cre recombinase, it was demonstrated that Lgr5 positive cells lie at the bottom of the crypt, intermingled with terminally differentiated Paneth cells. Most importantly, it was shown that such cells could differentiate into all four main cell types found within the intestinal epithelium meeting the definition of a stem cell. Counter-intuitively, Lgr5 cells are not nearly as quiescent, as stem cells would be expected to be, but divide every day. Lgr5 daughter cells constitute the transit-amplifying (TA) crypt compartment. TA cells divide every 12–16 h, generating some 300 cells per crypt every day; they reside within the crypts for approximately 48 h, undergoing up to five rounds of cell division while migrating upwards. When committed TA cells reach the crypt/villus junction, they rapidly differentiate into absorptive enterocytes, enteroendocrine cells or mucosecreting goblet cells, while continuing their upward migration. In contrast, Paneth cells escape this flow and reside for 3–6 weeks at the base of the crypt.

Despite the enthusiasm raised by the discovery of Lgr5 as the definitive marker of crypt stem cells, further research reignited the long lasting controversy over the exact location of intestinal stem cells. In fact, the exact identity of intestinal stem cells has proven controversial over the last 30 years, with two opposing models dominating the literature (80).

In the ‘+4 position’ model it was assumed that the base of the crypt is exclusively populated by terminally differentiated Paneth cells, and that stem cells should therefore be located just above Paneth cells at the +4 position. Instead, the more recent ‘stem cell zone’ model states that small, undifferentiated, cycling cells, intermingled with Paneth cells, are likely to be the true intestinal stem cells. These cells were termed crypt base columnar (CBC) cells, and are currently identified by the Lgr5 surface marker.

Moreover, in 2008, using an approach similar to the one implemented by Clevers *et al*, Sangiorgi and Capecchi identified Bmi1 as a further marker for adult intestinal stem cells (10). Remarkably, Bmi1-positive cells appear to occupy the previously described +4 position, and therefore are distinct from Lgr5 positive CBC cells. Moreover, whereas Lgr5 positive (Lgr5^+^) cells are known to be rapidly dividing, in most cases Bmi1 positive cells are quiescent or slowly replicating.

These findings have stimulated rethinking of the biology of the stem cell niche: distinct quiescent and active stem cell compartments may coexist within rapidly self-renewing tissues like the gut, skin and bone marrow, in separate yet adjoining locations. Actively dividing stem cell types (Lgr5^+^ cells in the intestine) could serve to maintain the regenerative capacities of these tissues under homeostatic conditions, whereas quiescent cells, less affected by environmental stresses (Bmi1^+^ in the intestine), could be held in reserve, playing the role of ‘backup’ cells (81).

There has been considerable discussion concerning whether cancer arises from adult stem cells. This issue has been thoroughly investigated in murine models of carcinogenesis in the small intestine. The contribution of adult intestinal stem cells to colorectal cancer initiation has been studied by transgenic induction of the conditional deletion of APC exclusively in the Lgr5^+^ intestinal stem cell compartment. This resulted in rapid transformation of Lgr5^+^ stem cells, which persisted and fuelled the growth of large adenomas mainly in the small intestine, but also in the colon. In contrast, and remarkably, APC deletion in non-stem TA cell populations failed to sustain significant adenoma growth (9).

The issue of the cell of origin of intestinal cancer was further investigated in quiescent intestinal stem cells marked as Bm1-positive (Bmi1^+^), where transgenic ectopic expression of an oncogenic variant of β-catenin was also found to promote intestinal neoplasia in mice (10). Overall, these observations support the hypothesis that stem cells are the predominant cell-of-origin of colorectal cancer.

Two models for the histogenesis of colorectal cancer in humans have been suggested: the ‘top-down’ model, proposing that dysplastic cells located in the intracryptal region eventually spread laterally and downwards to form new crypts, and the ‘bottom-up’ model, which posits that the inceptive cancer derives from the base of the crypt spreading upwards (82,83).

A number of findings in murine models of bowel tumorigenesis lend support to the bottom-up hypothesis for the generation of adenomas.

Whereas the outstanding, clear-cut results described above indicate that the main cell of origin for cancer in the small intestine of mice is the adult stem cell, the current, dynamic view of the cancer stem cell hypothesis, that somehow also applies to bowel cancer models, maintains that intestinal stem cells are not the only cells from which cancer may originate. It is now clear that cancer-propagating cells, *viz.* the cancer stem cells, may regenerate during neoplastic progression from TA to cancer cells, most notably during the epithelial-mesenchymal transition (EMT). In other words, the present view is that almost any somatic cell, normal or cancerous, has the potential to trigger or propagate the neoplasia in the presence of appropriate genetic or epigenetic conditions (84,85).

In continuously renewing ‘labile’ tissues, such as the epidermis, the gut and the bone marrow, it is now accepted that cancer arises mostly from adult, normal stem cells. However, the case of stable tissues (a most investigated example is the liver) is different, in that a wider range of cell types can be the target, and then the origin, of tumorigenesis (86).

## 5. The impact of pro-inflammatory signaling on bowel adult stem cells. Unanswered questions and possible experimental approaches

**Figure 2:**
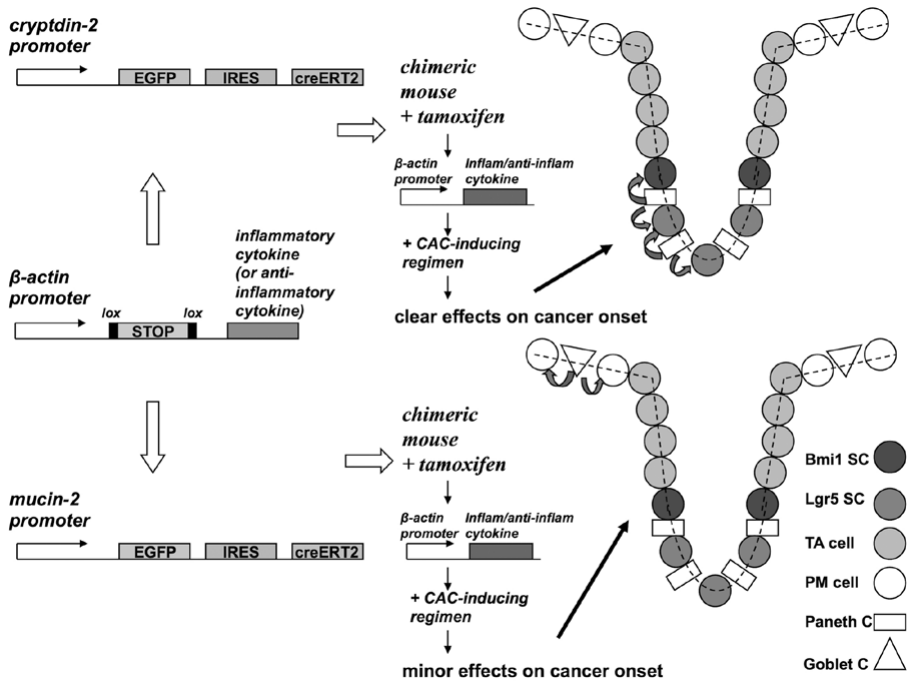
Investigation of intestine carcinogenesis by ectopic expression of pro/anti-inflammatory cytokine transgenes in Paneth cells. To obtain the spatial and temporal control of transgene expression, a *creERT2* construct encoding the cre recombinase fused to a tamoxifen-activated ERT2 mutant estrogen ligand binding domain may be used (87). A cell-specific promoter region [cryptdin-2 for Paneth cells (102) or mucin-2 for goblet cell (103)] drives the transcription of the fusion recombinase whereas the gene of interest (coding for a pro-inflammatory or an anti-inflammatory cytokine) is cloned downstream a housekeeping promoter (β-actin), but it is not expressed due to a loxP-flanked (floxed) STOP cassette. In chimeric mice that bear both trasgenes, exposure to tamoxifen may drive the cre-mediated deletion of the floxed STOP cassette, and hence the conditional expression of the cytokine in the cell of interest (i.e., Paneth or goblet cells). In this figure the direct targets of the ectopic cytokine are adjacent epithelial cells; alternatively, the action of the cytokine may be indirectly mediated through a stromal cell, most likely of myeloid lineage, as reported by Tu *et al* (95). Candidate pro-inflammatory or anti-inflammatory cytokines for transgenic expression may be TNFα or IL-10, respectively. In order to produce a substantial effect on cancer onset, this transgenic model should be coupled with a treatment predisposing to cancer in the intestine (e.g., a CAC regimen). In the figure stem and trans-amplifying cells are shown in different gray shades, whereas fully differentiated post-mitotic cells are shown in white.

In order to draw a more conclusive picture of the events linking inflammation to the early steps of oncogenesis in intestinal stem cells, the tumorigenic potential of ectopic, chronic release of pro-inflammatory cytokines in the adult intestinal stem cell niche should be investigated in detail. Specific *in vivo* models should be designed, to convey chronic inflammation in the close proximity of adult intestinal stem cells with the prospect of initiating neoplastic transformation. Targeted ectopic expression of pro-inflammatory cytokines in Paneth cells may offer the opportunity to redirect adult intestinal stem cells toward oncogenesis by acting directly on the stem cell niche. To illustrate this hypothesis a standard cre-lox tamoxifen-inducible strategy of transgenic expression in a cell of interest may be designed, as described in Fig. 2 (87).

We believe that an in-depth examination of the rationale and feasibility of such an experimental approach could raise a number of interesting issues regarding tumor biology in the intestine and, more broadly, the interplay between chronic inflammation and cancer stem cells.

In this regard, the following points seem particularly worthy of discussion:

- It is well known that in humans gut cancer is most frequently found in the colorectum. On the other hand, our intended target of transgenesis, i.e., the Paneth cell, is restricted to the crypts of the small intestine in both humans and mice. Consequently, in this case neoplasia is expected to be confined to the small intestine of mice. Actually, this circumstance does not constitute a difficulty for the experimental approach under scrutiny, as most murine models of bowel cancer show neoplastic growth chiefly in the small intestine (49,88–90).
  The pathophysiology of the gut is different in humans and mice, as demonstrated, for example, by the fact that when aged C57BL/6 mice were checked for spontaneous bowel cancer, the majority of the neoplastic lesions were located in the small intestine (91). Nevertheless, rodent models recapitulate quite faithfully the adenoma-carcinoma sequence that is found in human familial or sporadic CRC. Hence, murine models of intestine cancer have been always regarded as informative for human colon cancer (92).
- Paneth cells serve as multifunctional ‘guardians’ of crypt stem cells, both by secreting bactericidal products and by providing essential niche signals. This second feature of Paneth cells has been demonstrated recently: co-culturing of sorted intestinal stem cells with Paneth cells markedly improved *ex vivo* crypt formation, suggesting that the latter contributes to the construction of the stem cell niche (93,94).
  Experimental strategies should be aimed at ‘hijacking’ the crypt stem cell niche biology by transforming the function of the Paneth cell from *homeostasis keeper* to *local inducer of chronic inflammation*. To this end, the choice of the paracrine ectopic inflammatory stimulus is a relevant aspect. An obvious option could be an inflammatory cytokine though, to our knowledge, only a paper by Tu *et al* has reported the chronic, ectopic expression of an inflammatory cytokine in a cancer-prone epithelial layer of the gastrointestinal tract (95).
  The results of Tu *et al* suggest that the overexpression of a single cytokine, IL-1β, in the gastric mucosa may be sufficient to induce cancer in that site. Moreover, in that experiment tumorigenesis was triggered by a myeloid population recruited by IL-1β, suggesting that the inflammatory infiltrate contributed not only to cancer progression but also to the earlier stages of carcinogenesis. The important conclusion that can be drawn from this murine transgenic model is that in the bowel inflammation alone suffices for tumor development.
  For the choice of the inflammatory cytokine to be ectopically expressed in Paneth cells, IL-1β or TNFα could be interesting candidates, as *in vitro* experiments showed that these cytokines may over-stimulate in a paracrine fashion the Wnt/β-catenin pathway in colon carcinoma cell lines (41–43). Conversely, Paneth cell-targeted overexpression of an anti-inflammatory cytokine, such as IL-10, could create a ‘shield’, protecting the stem cell niche and attenuating the oncogenic potential of a CAC-inducing regimen on the whole mucosa. The fact that IL-10-deficient mice may spontaneously develop colitis, and subsequently colorectal tumors supports the use of this cytokine in this context (96).
- Another point worthy of investigation could be the most likely cell source of oncogenic inflammatory stimuli *in vivo*. While it is generally agreed that myeloid cells may play a role in this respect, it would be important to investigate whether IECs, or more specifically Paneth cells, may secrete significant amounts of inflammatory cytokines in healthy or diseased gut mucosal tissues. This is in fact the case of the preferential secretion by Paneth cells among IECs of TNFα leading to IBD, particularly Chron’s disease, and possibly to cancer, even if this last aspect has not been addressed (97).
  At present the issue of cytokine production and/or response by the epithelia lining the mucosal surface is rather ill-investigated. Some key questions for future investigation should be: what is known about secretion of cytokines by mucosal epithelial cells? Is this secretion directional? What is the identity of the cellular target of cytokines secreted by IECs, bystander cells or the same epithelial cells? What is the function, in healthy or diseased tissues, of cytokine secretion into mucosal fluids, for example, saliva?
- Another important aspect is that in most cases the published transgenic mouse models of gut cancer show an intrinsic low rate of cancer. Where this happens, in order to observe a significant increase of tumor incidence over the parental controls, it is mandatory to challenge the animals with a CAC-inducing regimen or to produce double transgenic mice. To this aim, a most widely used carcinogenic regimen consists of azoxymethane AOM, a colonotropic mutagen, followed by dextran sulfate sodium DSS, an intestine irritant (90).
- Finally, models of transgenic mice harboring targeted modification of goblet cells overexpressing inflammatory cytokines would be expected to display a low rate of gut cancer in animals exposed to a CAC-inducing regimen, since goblet cells in the epithelial monolayer lining the gut mucosa of the small intestine reside far outside the crypt niche (98).

## 6. Concluding remarks

At present, little is known about the roles of crypt stem cells in colitis-induced colon cancer, even in mice. The principal concern of this article is to hypothesize the likely outcome of a bowel ectopic stem cell niche redirected toward a pro-inflammatory state. By answering the questions that arise from the targeted trasgenesis of Paneth cells, a gain in comprehension could be achieved on the origin and pathogenesis of bowel cancer, even in humans.

It is hotly debated to what extent stemness is a cell-autonomous, intrinsic property of cells or, rather, the result of inputs coming from the intestinal niche (99). In this respect, the small intestine crypt represents an outstanding model, due to the unique role of Paneth cells in the fine-tuning of stem cell niche homeostasis. These cells secrete substantial quantities of anti-microbial peptides that protect the niche environment from the invasion by the gut microbiota. In addition and intriguingly, Paneth cells secrete factors that sustain and fuel the crypt stem cell compartment (93).

The idea of a targeted expression of pro- or anti-inflammatory cytokines in Paneth cells should be evaluated in the context of the evidence reviewed in the present article. In this regard, the construction of a ‘precancer niche’ has been hypothesized as a necessary early step required for initiated cells to survive and evolve toward cancer (100).

From the early reports by the Clevers’s lab, the widespread significance of Lgr5 as a stemness marker has been further endorsed. In mice, Lgr5 is a stem cell marker also in the crypt of the large intestine. Moreover, in humans, Lgr5 is a selective marker for human colorectal cancer stem cells (101).

It should be stressed that the results obtained in mice indicating Lgr5^+^ and/or Bmi1^+^ crypt stem cells as the cells-of-origin of bowel cancer most likely pertain to the familiar or sporadic CRC.

In conclusion, genetic studies with specific stem cell markers or the targeted trasgenesis of Paneth cells herein discussed should provide more information in the near future on the cell-of-origin of colitis-associated cancer in mice, but even in humans.
